# SOCIAL ISOLATION AND COGNITIVE FUNCTION: FOCUSING ON RACE/ETHNIC DIFFERENCES FROM THE NHATS STUDY

**DOI:** 10.1093/geroni/igad104.2733

**Published:** 2023-12-21

**Authors:** Murad Taani, Yura Lee, Julie Ellis, Chi Cho, Ammar Hammouri

**Affiliations:** University of Wisconsin-Milwaukee, Milwaukee, Wisconsin, United States; University of Wisconsin-Milwaukee, Milwaukee, Wisconsin, United States; University of Wisconsin Milwaukee, Milwaukee, Wisconsin, United States; University of Wisconsin Milwaukee, Milwaukee, Wisconsin, United States; University of Wisconsin Milwaukee, Milwaukee, Wisconsin, United States

## Abstract

Social isolation has negative associations with various health outcomes including cognitive function. However, limited research exists delineating the effect of social isolation on cognition using population-based longitudinal data. Moreover, little is known whether there are any race/ethnic differences in this social isolation-cognition relationship. We examined the effects of social isolation on cognitive function and explored the moderating role of race/ethnicity using longitudinal data of 4381 older adults from two waves of NHATS (2015 and 2018). Social isolation was measured by addressing four domains of network and integration: (a) marriage or partnership, (b) family and friends, (c) church participation, and (d) club participation (score range: 0 - 6). A higher score indicates more social isolation. Cognitive function was measured using the composite score of three domains: memory, orientation, and executive function (range: 0 - 33), with higher scores indicating better cognitive function. Repeated-measures ANOVA showed a significant relationship between social isolation and cognitive function. Specifically, the greater the social isolation, the lower the cognitive function. We found significant differences in cognitive function scores across race/ethnic groups. Non-Hispanic White showed higher cognitive function than non-Hispanic black, Hispanics, and other races. Non-Hispanic black and other races also showed significantly higher scores than Hispanics. The findings also showed that the cognitive function score significantly decreased over a 4- year period. There was no moderating effect of race/ethnicity found between social isolation and cognitive function. Interventions are needed to reduce social isolation by facilitating social participation in community activities, thereby protecting against cognitive decline.

